# Cytokine profiles, genetic polymorphisms, and systemic inflammatory markers in type 1 diabetes patients with COVID-19: IL-18 a predictor of disease severity

**DOI:** 10.3389/fimmu.2026.1736234

**Published:** 2026-03-02

**Authors:** Mohammed Yousif Abdullah, Ahmed Baligh Laaribi, Wafa Babay, Farah Abdulkhaleq Khattab Alhaddad, Asma Mehri, Hadda-Imene Ouzari, Sonia Marghali

**Affiliations:** 1 Laboratory of Microorganisms and Actives Biomolecules (LR03ES03), Faculty of Sciences of Tunis, University of Tunis El Manar, Tunis, Tunisia; 2 Medical Department, North Refineries Company, Ministry of Oil, Salahuddin, Iraq; 3 Department of Chemistry, Graduate School of Natural and Applied Sciences, Erciyes University, Kayseri, Türkiye; 4 Salahuddin Health Directorate, Ministry of Health, Salahuddin, Iraq

**Keywords:** cytokine profiles, COVID-19, inflammatory markers, IL-18, genetic polymorphisms, machine Learning, predictive biomarkers, T1DM

## Abstract

**Background:**

Patients with Type 1 Diabetes Mellitus (T1DM) are at increased risk for severe COVID-19 due to chronic inflammation, immune dysregulation, and impaired antiviral responses. However, the combined contribution of cytokine imbalance, host genetic variation, and systemic inflammation on COVID-19 severity in this population remains incompletely understood. This study aimed to investigate inflammatory biomarkers, interleukin profiles and immunogenetic factor associated with COVID -19 outcomes in T1DM patients.

**Methods:**

A total of 220 individuals were enrolled, including 160 T1DM patients with confirmed COVID-19 and 60 healthy controls. Serum concentrations of IL-6, IL-10, IL-12A, and IL-18 were quantified using ELISA, along with clinical inflammatory markers (CRP, D-dimer, and NLR). Genetic polymorphisms in the IL-10, IL-12A, IL-6, and IL-18 genes were analyzed using PCR-ARMS. Statistical analyses included group comparisons and correlation analysis. In addition, supervised machine-learning (ML) approaches (Decision Tree and Random Forest) were applied within the T1DM COVID-19 cohort to identify biomarkers predictive of disease severity.

**Results:**

Patients exhibited significantly elevated cytokine levels and inflammatory markers compared to controls (all p < 0.001). The IL-10 polymorphism (rs1800872) was significantly associated with increased disease susceptibility, with the G allele conferring a higher risk (p<0.0001, OR = 2.85 CI95% [1.83-4.43]). IL-12 correlated positively with IL-18 (r = 0.41, p < 0.001) and negatively with IL-6 (r = −0.22, p = 0.005). ML analyses identified IL-18, CRP, and IL-6 as the most informative biomarkers of COVID-19 severity, with IL-18 showing the highest feature importance (0.276, 0.202, and 0.175, respectively).

**Conclusion:**

This study highlights the critical role of inflammatory biomarkers and host genetic factors in COVID-19 severity among T1DM patients and identifies IL-18 as a robust and clinically relevant biomarker for risk stratification.

## Introduction

The global impact of coronavirus disease 2019 (COVID-19) has been particularly severe among individuals with pre-existing comorbidities, including Type 1 Diabetes Mellitus (T1DM) (1). Patients with T1DM are at increased risk for adverse COVID-19 outcomes due to impaired immune regulation, metabolic instability, and heightened susceptibility to complications such as diabetic ketoacidosis and hyperosmolarity (2). Epidemiological studies have consistently demonstrated higher rates of hospitalization, intensive care admission, and mortality among these patients compared with the general population (3, 4).

Disease severity in COVID-19 has been associated with dysregulated immune responses, characterized by elevated levels of pro-inflammatory cytokines, commonly referred to as the “cytokine storm” (5), including interleukin (IL)-6, IL-10, IL-12, IL-18, tumor necrosis factor-alpha (TNF-α), and interferon-gamma (IFN-γ) which have been correlated with poor clinical outcomes (6–8). Additionally, host genetic polymorphisms in immune-related genes may influence both cytokine expression and susceptibility to COVID-19 (9, 10). Furthermore, systemic inflammatory markers including C-reactive protein (CRP), ferritin, and D-dimer have also been identified as prognostic indicators of disease severity and patient survival (11).

Despite growing evidence linking immune dysregulation and host genetics to COVID-19 outcomes, few studies have simultaneously examined cytokine profiles, genetic polymorphisms, and inflammatory markers in T1DM patients infected with SARS-CoV-2. Given the unique immunometabolic context of T1DM, such an integrative approach is crucial to identifying predictors of disease severity and clinical outcomes.

The present study aims to investigate cytokine expression patterns (IL-6, IL-10, IL-12, and IL-18), selected genetic polymorphisms, and inflammatory markers in patients with T1DM infected with SARS-CoV-2. Specifically, we focus on functional single nucleotide polymorphisms (SNPs) within cytokine genes, including IL-6 (rs1800795), IL-10 (rs1800872), IL-12 (rs568408), and IL-18 (rs1946518), which have been linked to altered cytokine production and immune responses (12–14). These genetic variations have been implicated in increased susceptibility to severe infection, prolonged hospitalization, and poor survival outcomes (15, 16). By integrating immunological, genetic, and clinical data, this study seeks to identify potential biomarkers that can predict disease severity using machine learning (ML) model, ultimately contributing to improved risk stratification and personalized management strategies in T1DM patients with COVID-19.

## Materials and methods

### Study participants

This prospective observational study included 220 participants recruited between January 2022 and January 2023 from COVID-19 hospitalization centers operated by the Iraqi Ministry of Health. The study cohort comprised 160 patients diagnosed with T1DM and confirmed positive for SARS-CoV-2 by reverse transcription-polymerase chain reaction (RT-PCR) and were hospitalized in endocrinology departments, as well as 60 age- and sex-matched healthy individuals who served as controls.

Controls subjects were recruited during routine clinical screening or blood donation programs conducted within the same institutions. All controls tested negative for SARS-CoV-2 by RT-PCR at enrolment and had no history of COVID-19 infection. Individuals with metabolic disorders, inflammatory diseases, or acute infections were excluded from the healthy control group.

According to the World Health Organization (WHO) interim guidance for the clinical management of COVID-19, T1DM patients with COVID-19, were stratified into two subgroups: a mild/moderate group (n100) and a severe group (n=60)). Mild disease was defined by the presence of symptoms without evidence of pneumonia or hypoxia. Moderate disease was characterized by clinical signs of pneumonia (e.g., fever, cough, dyspnea, or fast breathing) without features of severe pneumonia. Severe disease was defined by the presence of at least one of the following criteria: oxygen saturation (SpO_2_) < 90% on room air, respiratory rate > 30 breaths/min, or signs of severe respiratory distress (17).

Written informed consent was obtained from all participants, and the study protocol was approved by the Research and Ethical Considerations Committee (RECC) of the Ministry of Health of Iraq.

### Inclusion and exclusion criteria

Eligible participants were adults aged 18 to 75 years. Patients in the T1DM group had a confirmed diagnosis of T1DM for at least one year in addition to laboratory-confirmed COVID-19 infection by RT-PCR. Individuals were excluded if they presented with autoimmune diseases other than T1DM, a history of malignancy or immunosuppressive therapy within the past six months, pregnancy or lactation, or incomplete medical records.

### Data collection

At admission, demographic and clinical information was recorded, including age, sex, body mass index (BMI), comorbidities (such as hypertension, cardiovascular disease, and obesity), and smoking status. Clinical assessment involved measurement of vital signs, including systolic and diastolic blood pressure, heart rate, and oxygen saturation. Hospitalization data, including length of stay, requirement for mechanical ventilation, and survival outcomes, were also documented.

### Inflammatory marker measurement

Systemic inflammatory markers were measured using standard laboratory procedures. CRP and D-dimer concentrations were quantified using the ichroma™ II analyzer (Boditech Med, South Korea) in accordance with the manufacturer’s instructions. The neutrophil-to-lymphocyte ratio (NLR) was calculated by dividing the absolute neutrophil count by the absolute lymphocyte count obtained from the complete blood count (CBC). All measurements were performed under standardized laboratory conditions to ensure analytical accuracy and reproducibility.

### Cytokine assays

Serum samples were collected from all participants within 24 hours of hospital admission and stored at -80 °C. Serum concentrations of cytokines were quantified using commercially available enzyme-linked immunosorbent assay (ELISA) kits according to the manufacturers’ protocols.

Interleukin-6 (IL-6) and interleukin-10 (IL-10) levels were measured using ELISA kits from BT Laboratory (Shanghai, China; catalog numbers E0090Hu and E0102Hu, respectively). The analytical sensitivity of the IL-6 assay was 1.03 pg/mL, while that of the IL-10 assay was 2.59 pg/mL. Interleukin-12 (IL-12) concentrations were determined using an ELISA kit from myBioscience (San Diego, CA, USA; catalog number MBS8801265), with a sensitivity of 3.3 pg/mL. Interleukin-18 (IL-18) levels were measured using a human IL-18 ELISA kit from Invitrogen™ (Thermo Fisher Scientific; product code 10079322), with a sensitivity of 6.25 pg/mL.

Optical density was measured using a microplate reader. Cytokine concentrations were calculated from standard curves generated using recombinant cytokine standards and were expressed in picograms per milliliter (pg/mL). All assays were performed in triplicate.

### DNA extraction and genotyping assay

Genomic DNA was extracted from whole blood collected in EDTA tubes using the QIAamp DNA Blood Mini Kit (Qiagen, Chatsworth, CA, USA) following the manufacturer’s instructions. DNA concentration and purity were assessed using a NanoDrop spectrophotometer (Thermo Fisher Scientific, USA).

Polymerase chain reaction (PCR) amplification was carried out in a final reaction volume of 25 µL containing 100 ng of genomic DNA, 12.5 µL of 2× Green Master Mix (Promega, USA), 0.75 µL of outer forward primer (0.4µM), 0.75 µL of outer reverse primer (0.4 µM), 1.5 µL of inner forward primer (0.2 µM), and 1.5 µL of inner reverse primer (0.2 µM) (**Table 1**).

**Table 1:** Specific primers of each polymorphism used in this study.

Gene	SNP	Primer sequence (5′-3′)	PCR	Amplicon size
IL-6	rs1800795 G>C	FI: CACTTTTCCCCCTAGTTGTGTCTTCCC	ARMS	206
		RI: ATTGTGCAATGTGACGTCCTTTAGCTTC		155
		FO: CAAGACATGCCAAAGTGCTGAGTCACT		306
		RO: AGAATGAGCCTCAGACATCTCCAGTCC		306
IL-10	rs1800872 G>T	FI: TACTTTCCAGAGACTGGCTTCCTACGGG	ARMS	211
		RI: GAACACATCCTGTGACCCCGCCTATA		260
		FO: CTAGGTCACAGTGACGTGGACAAATTG		417
		RO: GGTCATGGTGAGCACTACCTGACTAGC		417
IL-12A	rs568408 G>A	FI: GAAGGATGGGACTATTACATCCACCTA	ARMS	193
		RI: AAATGTCAAAAATACTTGATCAGAGGTC		136
		FO: CCAAACCGTTGTCATTTTTATAAAACTTT		272
		RO: CCACAAACACTTTGGTACAGAAATAGCT		272
IL-18	rs1946518 G>T	FI: GATACCATCATTAGAATTTTGTG	ARMS	204
		RI: GCAGAAAGTGTAAAAATTATCAA		278
		FO: CCTACAATGTTACAACACTTAAAAT		436
		RO: GCCCTAAATATATGTATCCTTAAAT		436

FI, Forward inner; RI, Reverse inner; FO, Forward outer; RO, Reverse outer.

Briefly, The thermal cycling conditions used for amplification of IL-6 (rs1800795), IL-10 (rs1800872), IL-12 (rs568408), and IL-18 (rs1946518) polymorphisms consisted of an initial denaturation at 95 °C for 2 minutes, followed by 35 cycles of denaturation at 95 °C for 20 seconds, annealing at 57 °C for 20 seconds, and extension at 72 °C for 1 minute, with a final extension step at 72 °C for 5 minutes.

**Figure 1:**
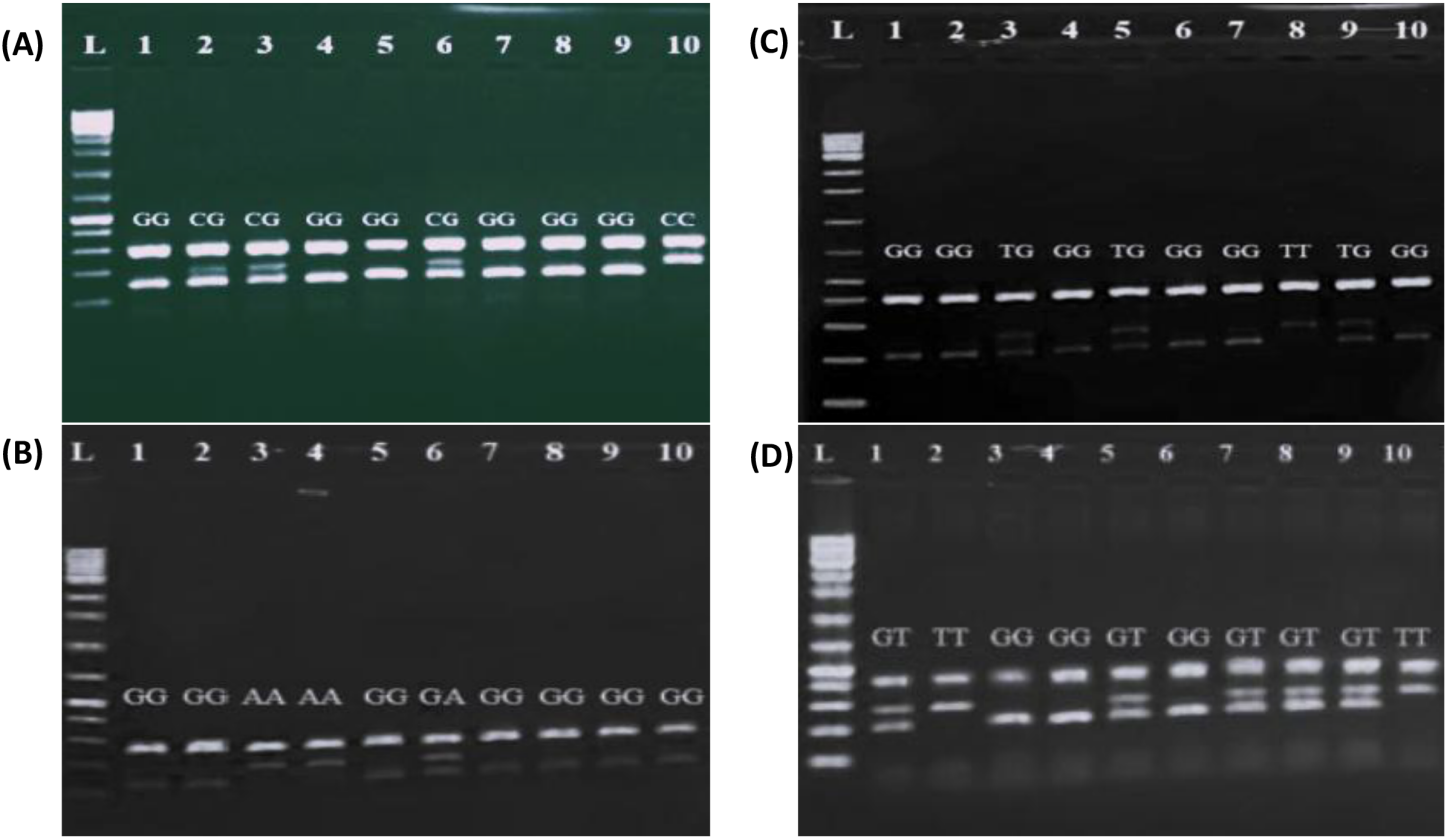
Representative agarose gel electrophoresis images showing PCR-ARMS genotyping of cytokine gene polymorphisms. **(A)** IL-6 SNP (rs1800795) genotyping: L= DNA ladder of 100 bp; GG genotype in lanes 1, 4,5, 7,8 and 9; CG genotype in lanes 2, 3, and 6; CC genotype in lane 10. **(B)** IL-10 SNP (rs1800872) genotyping: L= DNA ladder of 100 bp; GG genotype in lanes 1, 2, 4, 6, 7 and 10; TG genotyping in lanes 3, 5 and 9; TT genotyping in lane 8. **(C)** IL-12A SNP (rs568409) genotyping: GG genotyping in lanes 1, 2, 5, 7, 8, 9 and 10; GA genotype in lane 6; AA genotype in lanes 3 and 4. **(D)** IL-18 SNP (rs1946518): GG genotype in lanes 3, 4 and 6; GT genotype in lanes 1, 5, 7, 8 and 9.

PCR products were separated by electrophoresis on a 2% agarose gel stained with ethidium bromide, and visualized under ultraviolet illumination using a Gel Doc XR Plus system (Bio-Rad, USA) (**Figure 1**).

### Statistical analysis

All statistical analyses were performed using SPSS version 21.0 (SPSS Inc., Chicago, IL, USA) and GraphPad Prism 9 (GraphPad Software Inc., La Jolla, CA, USA). Quantitative data were summarized using descriptive statistics, with continuous variables expressed as mean ± standard deviation (SD), whereas non-normally distributed variables were expressed as median with interquartile range (IQR). Data normality was assessed using the Shapiro-Wilk test for all clinical and biochemical parameters, including interleukins (IL-6, IL-10, IL-12, IL-18), CRP, D-dimer, SpO_2_, and NLR. Correlation analyses were performed using Spearman’s rank correlation coefficient, as the data showed a non-normal distribution. Comparisons between patient and control groups were performed using the independent samples t-test for normally distributed variables and the Mann-Whitney U test for non-parametric data. To account for multiple comparisons across cytokines and inflammatory markers, p-values were adjusted using the Holm-Bonferroni correction. All statistical test were two-tailed, and p-value <0.05 was considered statistically significant.

Genotype and allele frequencies of cytokine gene polymorphisms were compared using the Chi-square test or Fisher’s exact test, as appropriate. Odds ratios (ORs) with 95% confidence intervals (CIs) were calculated to estimate relative risk. A p-value < 0.05 was considered statistically significant. A Bonferroni correction was applied for genotype comparisons.

### Machine-learning analysis

Supervised machine-learning analyses were performed exclusively within the cohort of T1DM patients (n = 160) to identify inflammatory biomarkers predictive of disease severity. Model inputs included seven clinically relevant inflammatory markers: IL-6, IL-10, IL-12, IL-18, CRP, D-dimer, and NLR. No *a priori* feature elimination was applied; instead, feature selection was implicitly handled through Gini impurity reduction in the Decision Tree model and mean decrease in Gini impurity in the Random Forest model.

The dataset was randomly split into training (70%) and testing (30%) subsets using stratified sampling to preserve class proportions. Class imbalance (mild/moderate n = 100 vs. severe n = 60) was addressed by applying inverse class-frequency weighting during model training. A Decision Tree classifier was constructed using the Gini impurity criterion with constrained maximum depth to limit overfitting and enhance interpretability. In parallel, a Random Forest classifier comprising 100 trees was implemented, with square-root feature sampling at each split, to improve predictive performance and model stability via bootstrap aggregation.

Model performance was evaluated on the independent test set using confusion-matrix–derived metrics, including sensitivity, specificity, and classification accuracy were derived from the confusion matrix, based on the numbers of true positives (TP), true negatives (TN), false positives (FP), and false negatives (FN). These terms were defined as follows:

- TP: T1DM patients with severe COVID-19 correctly classified as severe.
- FP: T1DM patients with mild/moderate COVID-19 incorrectly classified as severe.
- TN: T1DM patients with mild/moderate COVID-19 correctly classified as mild/moderate.
- FN: T1DM patients with severe COVID-19 incorrectly classified as mild/moderate.

These metrics were used to evaluate model performance on the independent test set.

## Results

### Demographic and clinical characteristics

The study involved 220 participants divided into two groups: 160 patients with T1DM and COVID-19, and 60 healthy controls. The mean age was 55.66 ± 8.70 years in the patient group and 54.82 ± 8.55 years in the control group, showing no statistically significant difference (p = 0.523). Body Mass Index (BMI) analysis revealed that a higher proportion of patients fell into the overweight and obese categories compared to the control group (**Table 2**).

**Table 2:** Demographic and clinical characteristics.

Characteristic	Control group	Patient group	*P-value*
Age (years)	54.82 ± 8.55	55.66 ± 8.70	ns
Sex (M/F)	35/25	90/70	ns
BMI (kg/m²)	24.5 ± 2.5	28.3 ± 3.2	<0.001
Underweight (<18.5)	2 (3.3%)	5 (3.1%)	ns
Normal (18.5–24.9)	30 (50%)	45 (28.1%)	<0.001
Overweight (25.0–29.9)	20 (33.3%)	60 (37.5%)	ns
Obese (≥30)	8 (13.3%)	50 (31.3%)	<0.001
ALT	17.15± 4.6	67.88 ± 23.66	<0.001
AST	21.28 ± 3.79	41.26 ± 7.06	<0.001
ALP	82.95 ± 20.36	100.5 ± 25.31	<0.001

ns, no significant.

### Serum cytokine profiles and inflammatory biomarker levels

**Figure 2:**
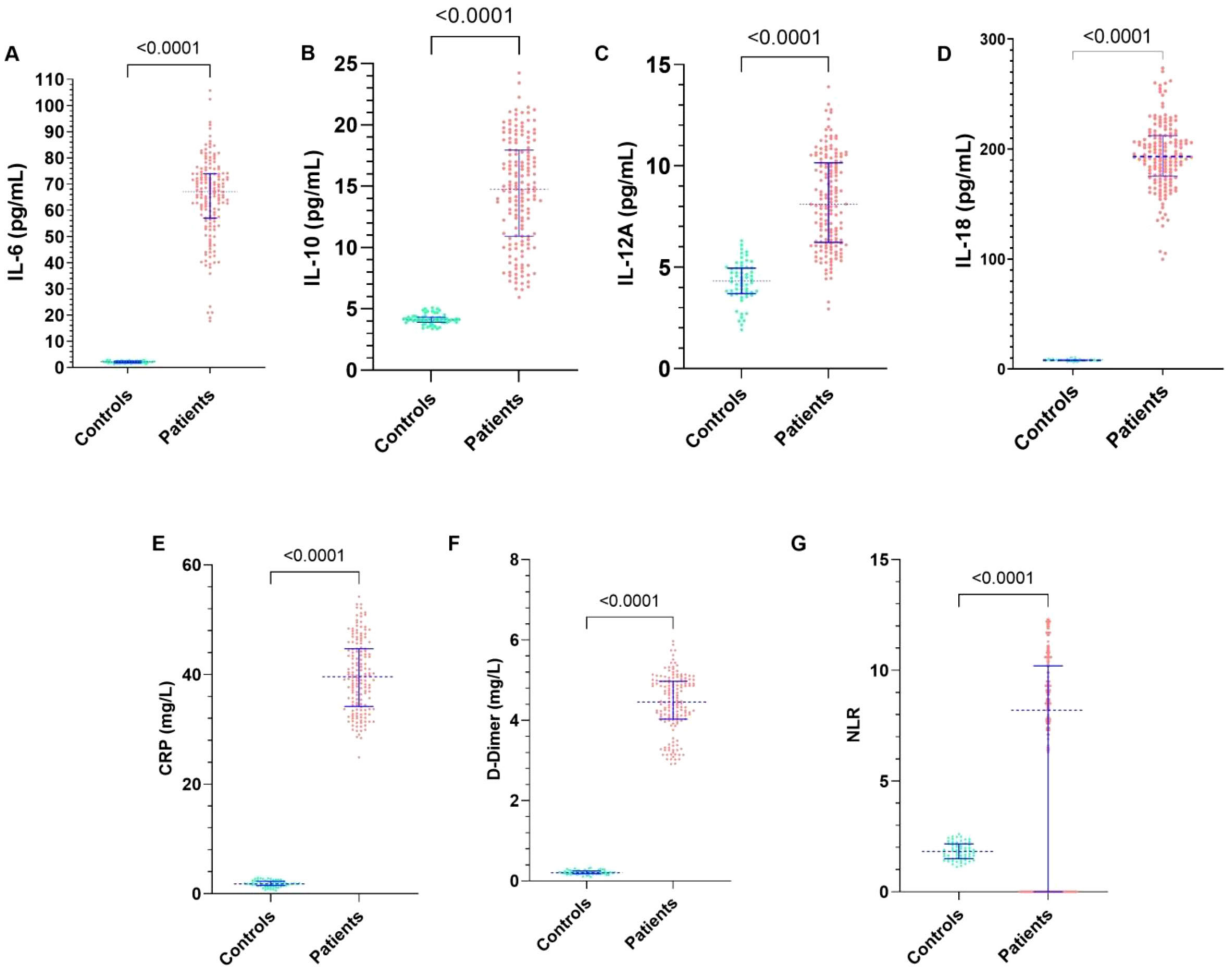
Comparison of serum cytokine, inflammatory markers and haematological indices between patient s and healthy controls. Serum concentrations of **(A)** IL-6, **(B)** IL-10, **(C)** IL-12A, **(D)** IL-18, **(E)** C-reactive protein (CRP), and **(F)** D-dimer were measured using standard immunoassays, while **(G)** the neutrophil-to-lymphocyte ratio (NLR) was calculated from complete blood count data. Each dot represents an individual value, and horizontal lines indicate the median ± IQR. All markers were significantly higher in patients compared with healthy controls (*p* < 0.0001 for all comparisons, Mann–Whitney U test).

As shown in **Figure 2**, inflammatory and immunological biomarkers are presented as median with IQR. All measured biomarkers showed significant differences between patients and controls (Mann-Whitney U test, all p < 0.0001), and these associations remained statistically significant after Holm–Bonferroni correction for multiple comparisons (α=0.0071).

Serum levels of IL-6 (**Figure 2A**), IL-10 (**Figure 2B**), IL-12A (**Figure 2C**), and IL-18 (**Figure 2D**) were markedly increased in patients compared with controls (IL-6: 67.03 [57.03-73.91] vs. 2.11 [1.73-2.45] pg/mL; IL-10: 14.77 [10.92-17.95] *vs*. 4.11 [3.9-4.32] pg/mL; IL-12: 8.1 [6.22-10.15] *vs*. 4.31 [3.69-4.95] pg/mL; IL-18: 193.3 [175.5-212.3] *vs*. 8.09 [7.6-8.5] pg/mL), indicating a pronounced pro-inflammatory and immunoregulatory activation in the patient group.

Similarly, levels of CRP (**Figure 2E**) and D-dimer (**Figure 2F**) were substantially higher in patients than in controls (CRP: 39.6 [34.18-44.70] *vs*. 1.8 [1.5-2.27] mg/L; D-Dimer: 4.45 [4.03-4.97] *vs*. 0.20 [0.18-0.24] mg/L), reflecting systemic inflammation and activation of coagulation pathway.

Furthermore, the NLR (**Figure 2G**) was significantly increased in patients compared with controls (8.2 [0-10.2] *vs*. 1.82 [1.49-2.16]; p < 0.001), underscoring its potential role as an indicator of immune imbalance dysregulation and disease severity. Collectively, these findings reveal a distinct inflammatory and immunological signature in affected individuals relative to healthy subjects.

### Correlation analysis between cytokine and inflammatory markers

**Figure 3:**
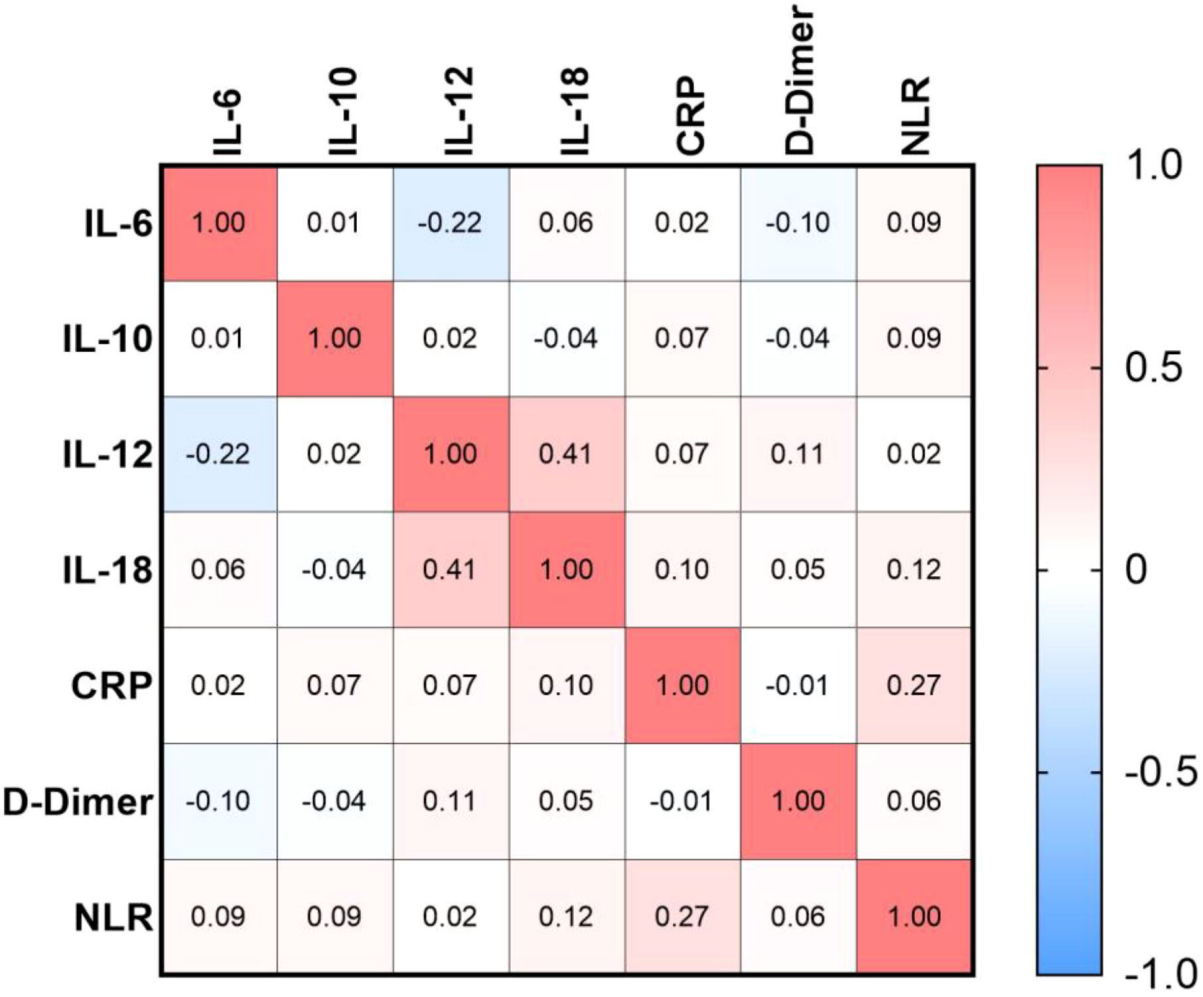
Correlation patterns among cytokines and inflammation-related biomarkers. The heatmap illustrates the pairwise correlation coefficients (Spearman’s r) among interleukins (IL-6, IL-10, IL-12, IL-18), inflammatory markers (CRP and D-dimer), and the neutrophil-to-lymphocyte ratio (NLR). Positive correlations are represented in red, whereas negative correlations are shown in blue, with color intensity reflecting the strength of association.

**Figure 3** illustrates the Spearman correlation coefficients (r) among the studied biomarkers in comorbid T1DM and COVID-19.

A moderate positive correlation was observed between IL-12 and IL-18 (r = 0.41, p<0.001), suggesting a coordinated pro-inflammatory immune response. In contrast, IL-12 exhibited a weak negative correlation with IL-6 (r = -0.22, p=0.005), indicating a potential regulatory divergence within cytokine signaling pathways.

Additionally, CRP showed a weak positive association with NLR (r = 0.27, p=0.001), supporting a possible link between systemic inflammation and hematological alterations. Overall, the correlation matrix underscores the complex interplay between cytokine levels and systemic inflammatory responses in patients.

### Association between cytokine gene polymorphisms and disease susceptibility

The genotypic and allelic distributions of IL-6 (rs1800795), IL-10 (rs1800872), IL-12 (rs568408), and IL-18 (rs1946518) polymorphisms were compared between COVID-19 patients with T1DM and healthy controls (**Table 3**). No significant differences were observed in either allele or genotype frequencies for IL-6 rs1800795 and IL-12 rs568408 polymorphisms (p > 0.05), suggesting no direct association with disease susceptibility.

**Table 3:** Genotype distribution and allele frequencies of Cytokines polymorphisms between patients and healthy controls.

SNP		Patients N=160 (%)	Controls N=60 (%)	P-value	P_Corrected_	OR [95%]
IL-6 (rs1800795)						
**Alleles**	**G**	172 (54)	62 (52)	0.69	0.77	1.08 [0.71-1.65]
	C	148 (46)	58 (48)	‡	‡	‡
**Genotypes**	**GG**	47 (29)	18 (30)	0.66	0.82	1.19 [0.53-2.66]
	GC	78 (49)	26 (43)	0.40	0.51	1.37 [0.65-2.87]
	CC	35 (22)	16 (27)	‡	‡	‡
IL-10 (rs1800872)						
**Alleles**	**G**	243(76)	63 (52)	1.9 10^-6^	3.4 10^-6^	2.85 [1.83-4.43]
	T	77 (24)	57 (48)	‡	‡	‡
**Genotypes**	**GG**	104 (65)	17 (28)	0.0007	0.0016	4.07 [1.74 -9.52]
	TG	35 (22)	29 (48)	0.61	0.76	0.8 [0.34-1.85]
	TT	21 (13)	14 (24)	‡	‡	‡
IL-12 (rs568408)						
**Alleles**	**A**	87 (27)	29 (24)	0.52	0.6	1.17 [0.72-1.9]
	G	233(73)	91 (76)	‡	‡	‡
**Genotypes**	**AA**	18 (11)	8 (13)	1^*^	1	0.96 [0.38-2.4]
	GA	51 (32)	13 (22)	0.15	0.20	1.68 [0.82-3.4]
	GG	91 (57)	39 (65)	‡	‡	‡
IL-18 (rs1946518)						
**Alleles**	**G**	142 (44)	46 (39)	0.25	0.3	0.77 [0.5-1.19]
	T	178 (56)	74 (61)	‡	‡	‡
**Genotypes**	**GG**	27 (17)	4 (7)	0.12*	0.15	2.7 [0.82-8.82]
	GT	88 (55)	38 (53)	0.82	0.95	0.92 [0.47-1.8]
	TT	45 (28)	18 (30)	‡	‡	‡

Risk alleles marked in bold letters; ‡: Reference genotype. *Fisher Exact test.

In contrast, a significant association was identified for IL-10 (rs1800872), with both allelic and genotypic distributions differing markedly between patients and controls (p < 0.001, which remained significant after correction for multiple comparisons, α = 0.0125). The G allele was significantly more frequent in patients (76%) than in controls (52%) and was associated with increased susceptibility to COVID-19 in T1DM patients (OR = 2.85; 95% CI = 1.83 - 4.43). Furthermore, the GG genotype predominated among patients (65%) compared with controls (28%) and showed a strong association with disease susceptibility (OR = 4.07; 95% CI = 1.74-9.52). In contrast, the TT and TG genotypes were significantly more prevalent in healthy individuals but did not reach a statistical significance.

For IL-18 (rs1946518), the GG genotype was more prevalent among patients (17%) compared with controls (7%). However, this distribution did not translate into a statistically significant association with disease susceptibility.

### IL-18 as a key predictive biomarker of disease severity

**Figure 4:**
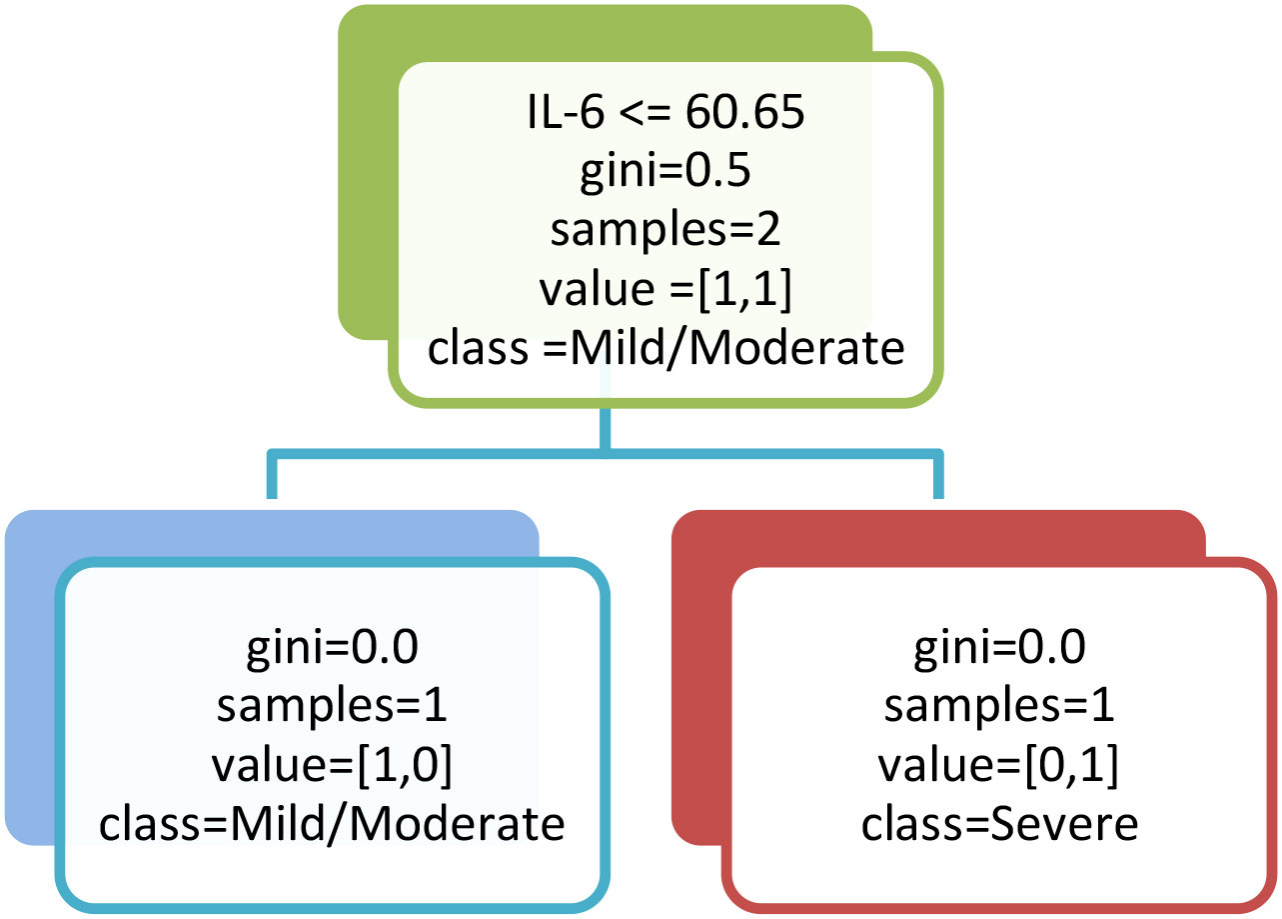
Decision tree model for classifying COVID-19 severity in T1DM patients based on inflammatory biomarkers. This figure illustrates a simplified representation of the decision tree used to discriminate between mild/moderate and severe COVID-19 cases. IL-6 was identified as the primary splitting variable, with a threshold of 60.65 pg/mL serving as the main decision boundary, effectively separating patients according to disease severity.

**Figure 5:**
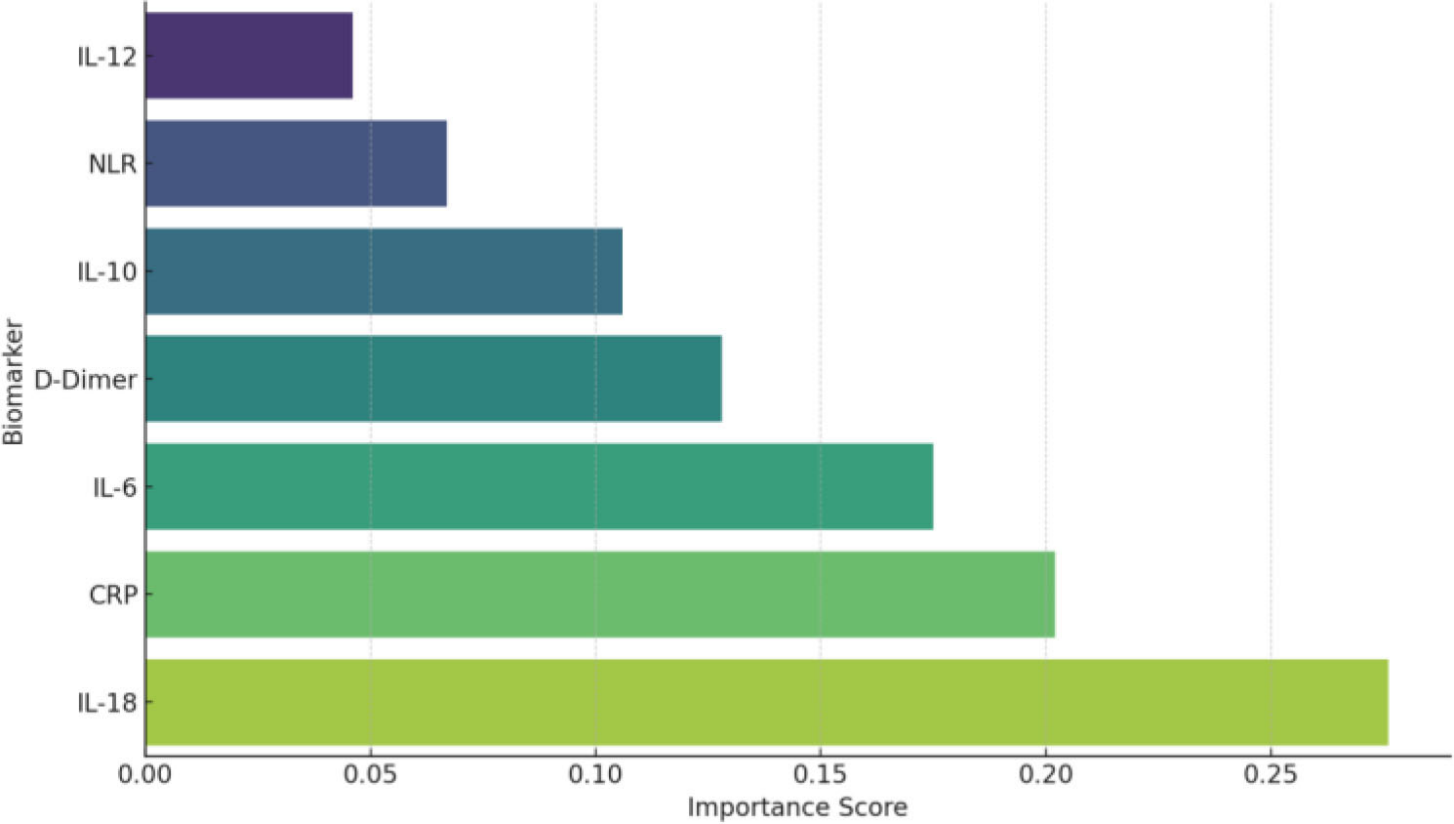
Feature importance plot (random forest).

To further investigate the predictive power of inflammatory and genetic biomarkers for classifying COVID-19 severity in patients with T1DM, we employed supervised machine learning models (**Figure 4**). A Decision Tree classifier, using key immunological variables, selected IL-6 as the primary splitting variable at the root node. This selection reflects the optimal reduction in Gini impurity at the initial decision step rather than overall predictor dominance. The Random Forest model, which evaluates variable importance across multiple trees, consistently ranked IL-18 as the most significant biomarker at the root node, followed by CRP and IL-6. This model achieved an overall accuracy of 85.6%, with a sensitivity of 88.3% and a specificity of 81.7%. To enhance performance and mitigate overfitting, a Random Forest classifier, an ensemble of 100 trees, was subsequently implemented. This model demonstrated superior predictive power, yielding an accuracy of 91.2%, a sensitivity of 92.5%, a specificity of 89.1%, and a mean AUC of 0.975. Feature importance analysis from the Random Forest confirmed and reinforced the initial findings, ranking IL-18 (0.276), CRP (0.202), and IL-6 (0.175) as the top three predictors (**Figure 5**). These results underscore the critical role of hyperinflammatory markers, particularly IL-18 and CRP, in stratifying COVID-19 risk in T1DM patients and highlight the potential of ensemble methods like Random Forest as robust tools for clinical decision-support systems.

### Analysis of inflammatory biomarkers across COVID-19 severity subgroups in T1DM patients

Comparative analysis of key inflammatory biomarkers revealed significantly elevated serum levels in T1DM patients with severe COVID-19 compared to those with presenting mild/moderate disease. As shown in **Table 4**, IL-18 concentrations were markedly higher in the severe group (median [IQR]: (218 [208.4-230] pg/mL *vs*. 181.9 [165.8-192.3] pg/mL; p<0.0001), as were CRP (46.7 [44.2-48.7] mg/L *vs*. 35.7 [32.33-39] mg/L; p<0.0001) and IL-6 (76.23 [68.28-78.5] pg/mL *vs*. 60.76 [42.85-59.48] pg/mL; p<0.0001). All associations remained statistically significant after adjustment for multiple comparisons using the Holm–Bonferroni correction.

**Table 4:** Comparison of key inflammatory biomarker levels between patients with mild/moderate and severe COVID-19.

Marker	Mild/Moderate (n=100)		Severe (n=60)		p-value
	Median	IQR	Median	IQR	
IL-18 (pg/mL)	181.9	164.8-192.3	218	208.4-230	<0.0001
CRP (mg/L)	35.7	32.33-39	46.7	44.2-48.7	<0.0001
IL-6 (pg/mL)	60.76	42.85-59.48	76.23	68.28-78.5	<0.0001

These univariate findings were further validated using supervised machine-learning approaches: a Decision Tree classifier identified IL-18 as the primary node for disease severity stratification. Consistently a Random Forest model ranked IL-18, CRP, and IL-6 as the three most influential predictors of severe disease. The concordance between elevated serum biomarker levels and their high predictive importance underscores the critical role of this hyperinflammatory triad in determining COVID-19 severity among T1DM patients.

## Discussion

This study aimed to investigate cytokine dysregulation, genetic polymorphisms, and systemic inflammatory responses underlying disease severity and progression in T1DM patients affected by COVID-19, and to identify key biomarkers associated with clinical severity using machine learning approaches. Our findings highlight the complex interplay between host genetic background and immune activation, contributing to the observed heterogeneity in clinical outcomes among COVID-19 patients. The results underscore the combined impact of inflammatory cytokines and biomarkers (IL-6, IL-10, IL-12, IL-18, CRP, D-Dimer and NLR), alongside cytokine gene polymorphisms, in modulating the host response to SARS-CoV-2 infection.

In this study, T1DM patients with COVID-19 exhibited significantly elevated levels of IL-6, IL-10, IL-12, and IL-18 compared to healthy controls. These findings are consistent with prior reports linking cytokine hypersecretion to severe disease progression (8, 18, 19).

Among these cytokines, IL-6 has emerged as a pivotal mediator of COVID-19–associated hyperinflammation. It promotes T-helper 17 (Th17) cell differentiation and amplifies the release of other pro-inflammatory cytokines, thereby exacerbating systemic inflammation (20). Our results further support previous observations by Khafaei et al. (20) and Wang et al. (21), which correlated elevated IL-6 levels with higher mortality in severe cases. Interestingly, we also observed increased IL-10 levels in severe COVID-19 cases, reflecting a paradoxical immune response. Although IL-10 is classically anti-inflammatory, its elevation may represent a compensatory attempt to counterbalance hyperinflammation, a mechanism that ultimately appears insufficient to prevent systemic immune dysregulation (22). This dual pattern underscores the complex interplay between pro- and anti-inflammatory pathways in severe COVID-19, particularly in patients with underlying T1DM.

Elevated IL-18 levels observed in our cohort emphasize its role in severe COVID-19. Consistent with prior reports by Marino et al. (23) and Nasser et al. (24), IL-18 has been identified as a predictor of disease severity. This cytokine plays a critical role in activating natural killer (NK) cells and promoting IFN-γ production, thereby amplifying the inflammatory cascade in critically ill patients (25). The concurrent elevation of IL-18 alongside IL-6, IL-10, and IL-12 in T1DM patients with COVID-19 underscores a coordinated yet dysregulated immune response, likely contributing to the heightened susceptibility to severe outcomes in this population (10).

Genetic predisposition is increasingly recognized as an important determinant of individual susceptibility to COVID-19 infection and disease progression (26, 27). In this study, we identified significant associations between specific SNPs in cytokine-related genes and COVID-19 susceptibility, highlighting the potential contribution of host genetic factors in shaping immune responses to SARS-CoV-2 infection.

Notably, the IL-10 rs1800872 genotype was significantly associated with an increased susceptibility to COVID-19 infection. These findings are consistent with previous reports (28, 29), suggesting that this polymorphism may influence disease outcomes by modulating cytokine expression. The IL-10 rs1800872 variant has been linked to reduced anti-inflammatory activity, resulting in impaired regulation of pro-inflammatory cytokines and a subsequent exacerbation of the inflammatory response (29).

In contrast, the IL-6 (rs1800795), IL-12 (rs568408), and IL-18 (rs1946518) polymorphisms did not reach statistical significance in our cohort. Likewise, previous investigations by Falahi et al. (30), Benmansour et al. (31) and Smail et al. (32) have also reported no significant association with COVID-19 susceptibility. Nevertheless, accumulating evidence indicates that these SNPs may influence the course of various infectious diseases, including SARS-CoV-2 (12, 33), and HBV infections (34, 35). By modulating cytokine gene transcription and expression (36–38), these variants may shift the delicate balance between pro- and anti-inflammatory immune pathways, ultimately impacting viral clearance and tissue damage during SARS-CoV-2 infection.

The systemic inflammatory markers CRP, D-dimer, and NLR, were significantly elevated in our patients, reinforcing their established role as reliable prognostic biomarkers (39, 40). In this study, ML analyses identified CRP as one of the most powerful predictors of disease severity, which aligns with its correlation with systemic inflammation driven by an excessive immune response (40). Moreover, consistent with previous findings by Benotmane et al., CRP emerged as an independent predictor of adverse clinical outcomes, particularly among individuals with pre-existing comorbidities such as diabetes and cardiovascular disease (41).

Furthermore, ML analyses identified IL-18 as a key inflammatory mediator linked to COVID-19 severity, beyond classical inflammatory markers. IL-18 is a potent cytokine that promotes NK-cell activation and IFN-γ production, thereby amplifying the Th1-driven immune response. Elevated IL-18 levels observed in severe cases reflect an exaggerated innate immune activation, likely driven by inflammasome (NLRP3) hyperactivation during SARS-CoV-2 infection (42). Moreover, IL-18 levels were significantly correlated with IL-12, suggested its contribution to systemic inflammation and immune dysregulation in our patients. These findings align with Marino et al., who demonstrated significantly higher IL-18 levels in hospitalized COVID-19 patients and reported impaired regulation of IL-18-binding protein (IL-18BP) (23). Moreover, Maaß et al. identified IL-18 as an independent predictor of mortality, confirming its prognostic value in severe COVID-19. Persistent elevation of IL-18 has also been associated with tissue damage, endothelial dysfunction, and cytokine storm development in critical cases (43).

Based on the data generated in this study, IL-18 may be suggested as a potential marker associated with disease progression, particularly in high-risk individuals such as patients with diabetes. Dysregulated IL-18 signaling may contribute to the inflammatory milieu and immune dysregulation observed in severe COVID-19 in the context of metabolic disorders. Taken together, these findings suggest that IL-18 may play a contributory role in COVID-19 pathogenesis rather than acting as a primary driver in T1DM patients.

## Conclusion

In summary, this study highlights the role of several immune-related factors, including genetic variants, cytokines, and inflammatory markers, in contributing to COVID-19 susceptibility and disease progression in patients with T1DM. Elevated circulating cytokines, particularly IL-6, IL-10, IL-12, and IL-18, reflect a pronounced inflammatory imbalance that predisposes diabetic individuals to heightened disease severity. Among these mediators, ML based analysis showed IL-18 emerged as a central driver of hyperinflammation in this cohort. In parallel, the association between IL-10 rs1800872 polymorphism and increased disease susceptibility underscores the role of host genetics in shaping immune responses to SARS-CoV-2. Although other cytokine-related SNPs did not reach significance in our cohort.

## Limitations

This study has certain limitations that should be acknowledged. First, the control group consisted of healthy individuals without T1DM or COVID-19; therefore, the present design does not allow for a clear separation of the respective effects of T1DM and SARS-CoV-2 infection on immune and inflammatory responses. The absence of comparator groups, such as T1DM patients without COVID-19 or non-diabetic patients with COVID-19, limits the ability to disentangle disease-specific contributions. Second, the lack of significant genetic associations for certain polymorphisms, as well as discrepancies with findings from other studies, may be attributable to ethnic variability and sample size limitations. Larger, multiethnic cohorts will be necessary to validate these observations. Finally, although machine learning–based analysis was employed to explore key biomarkers associated with disease severity, the models were internally derived and lacked external validation. Future studies incorporating independent validation datasets and robust cross-validation strategies are warranted to enhance the reliability and translational relevance of these findings.

Continued research in diverse populations will be essential to optimize personalized approaches and improve outcomes in high-risk patients such as those with diabetes.

## Data Availability

The raw data supporting the conclusions of this article will be made available by the authors, without undue reservation.
